# Isolated Right Chylothorax Secondary to Superior Vena Cava (SVC) Obstruction Successfully Treated With Endovascular Stenting

**DOI:** 10.7759/cureus.86212

**Published:** 2025-06-17

**Authors:** Sridhar V Prabhu, Yugandhar S, Mithilesh Arumulla, Ravindra Adimulam, Vikram Damaraju

**Affiliations:** 1 Department of Radiology, All India Institute of Medical Sciences, Mangalagiri, Mangalagiri, IND; 2 Department of Pulmonology, All India Institute of Medical Sciences, Mangalagiri, Mangalagiri, IND

**Keywords:** chylothorax, endovascular stenting, interventional radiology, right sided chylothorax, superior vena cava obstruction, superior vena cava (svc) syndrome

## Abstract

Chylothorax, an uncommon cause of pleural effusion, typically results from thoracic duct injury or obstruction. Superior vena cava (SVC) obstruction is a rare etiology, often leading to right-sided chylothorax due to disrupted lymphatic drainage.

This case report describes a 70-year-old female patient with a history of breast carcinoma who presented with right-sided chylothorax secondary to SVC thrombosis caused by a neglected chemoport. A CT venogram confirmed chronic SVC occlusion. Endovascular intervention, including angioplasty and stenting, successfully restored venous patency, resolving the chylothorax within 48 hours. This case underscores the role of interventional radiology in diagnosing and treating central venous obstruction-related chylothorax, offering a minimally invasive and effective therapeutic approach.

## Introduction

Chylothorax, a rare cause of pleural effusion, arises from the accumulation of lymphatic fluid in the pleural space secondary to disruption or obstruction of the thoracic duct [1]. Common causes include direct injury to the thoracic duct following surgery or tumor infiltration, whereas superior vena cava (SVC) obstruction is a rare cause [2].

While most cases are left-sided due to the thoracic duct’s drainage into the left venous angle, right-sided chylothorax occurs when obstruction at the right internal jugular-subclavian junction or SVC disrupts normal lymphatic drainage from the right thoracic duct [2]. Central venous obstruction can lead to venous hypertension, lymphatic congestion, and persistent chylous effusion [3]. Endovascular SVC stenting offers a minimally invasive approach to restore venous and lymphatic flow [4].

## Case presentation

A 70-year-old female patient with a history of breast carcinoma, post-chemotherapy, radiotherapy, and surgery, presented with progressive dyspnea. Initial evaluation at an outside hospital revealed a right pleural effusion. She was subsequently referred to our center for further management. During a comprehensive evaluation, the patient disclosed a neglected right-sided chemoport that had remained in situ for three years and was removed three months ago.

**Figure 1:**
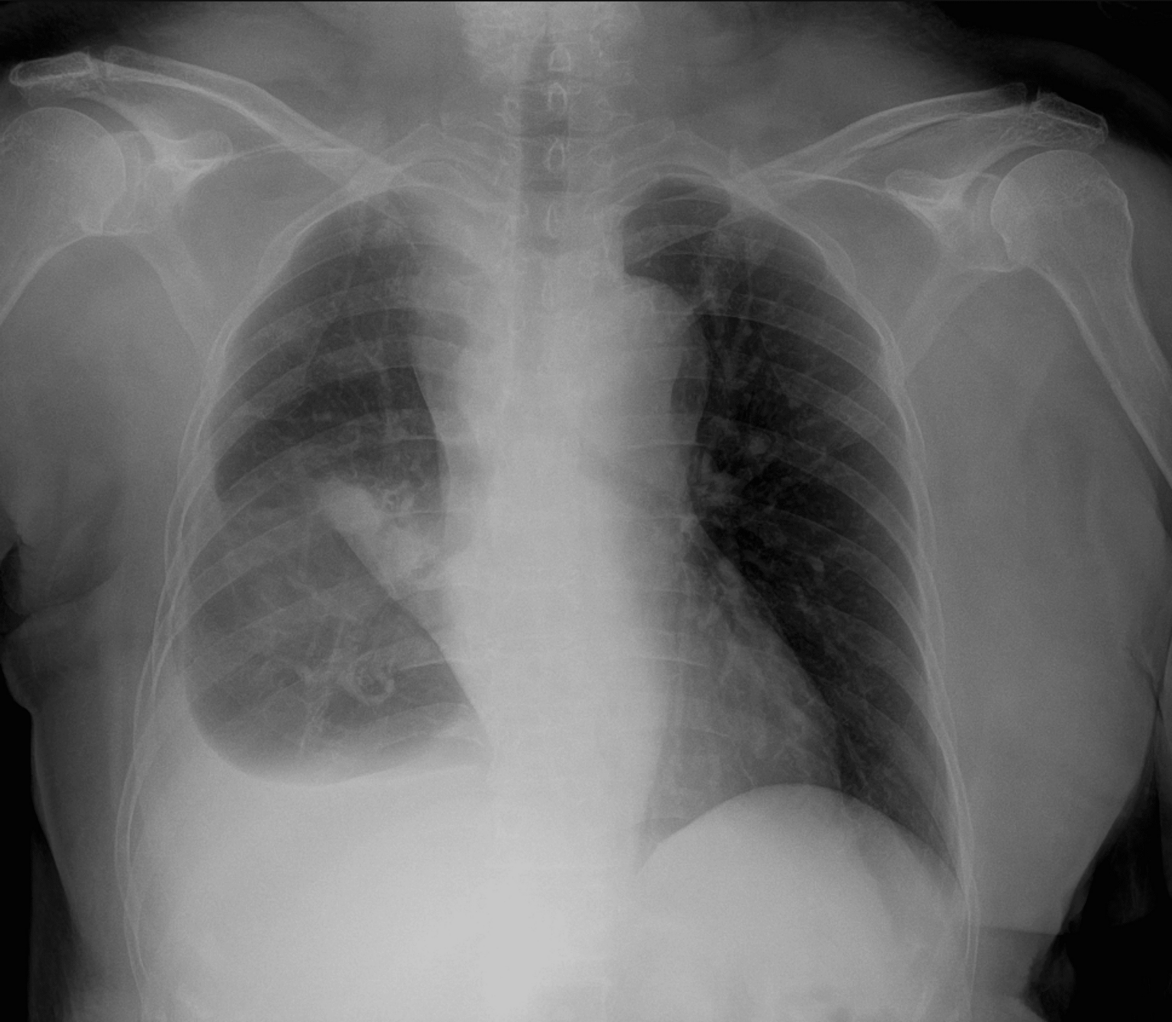
Pre-procedural frontal chest radiograph showing right pleural effusion.

At our institution, chest imaging confirmed a right-sided effusion (Figure 1), and thoracentesis revealed chylous fluid with negative cytology and microbiological studies. Fluorodeoxyglucose (FDG) PET-CT ruled out residual malignancy, further supporting a non-malignant cause for the effusion.

**Figure 2:**
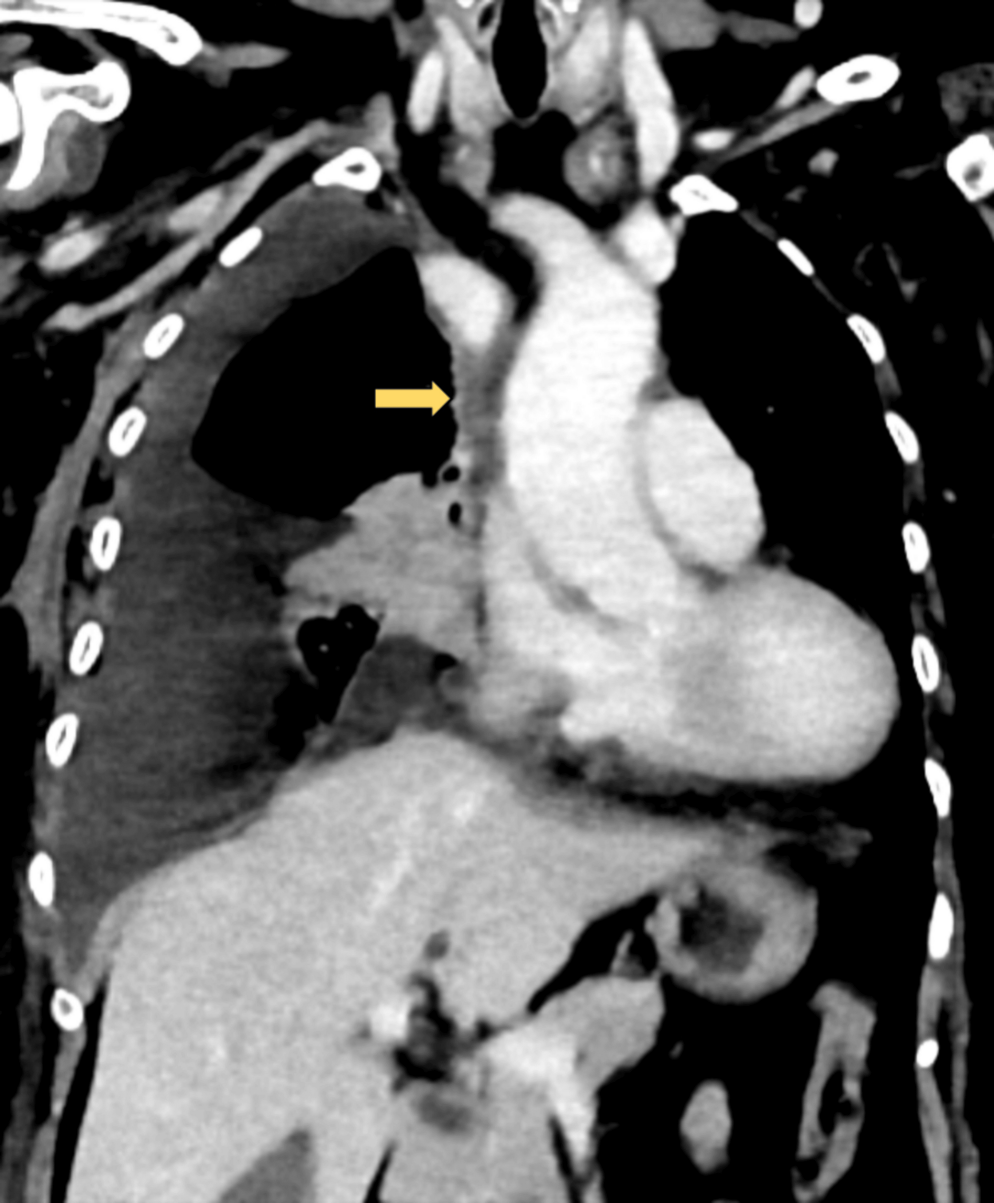
CT venogram demonstrating short segment (~3.3 cm) superior vena cava occlusion with luminal attenuation (arrow) and associated moderate right pleural effusion, subsequently confirmed as chylothorax.

Given the persistent chylous effusion, an interventional radiology consultation was requested. Due to the isolated right-sided nature of the effusion, central venous obstruction was considered a potential underlying cause, and a CT venogram of the chest was performed. The CT venogram demonstrated short-segment (~3.3 cm) SVC thrombosis with reduced luminal caliber, wall thickening, and absence of contrast opacification, along with prominent collateral vessels, findings consistent with chronic occlusion (Figure 2).

**Figure 3:**
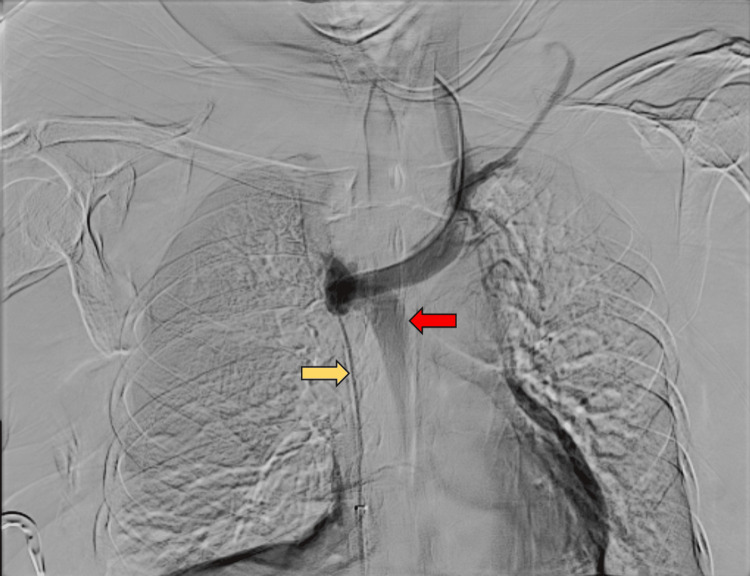
Digital subtraction venogram demonstrating short segment superior vena cava obstruction (yellow arrow) with collateral rerouting through the azygos vein (red arrow).

Endovascular intervention was deemed necessary. The right common femoral vein access was obtained, and digital subtraction venography demonstrated a short-segment SVC obstruction with collateral rerouting through the azygos vein (Figure 3). The obstruction was negotiated using a 0.035-inch hydrophilic guidewire.

**Figure 4:**
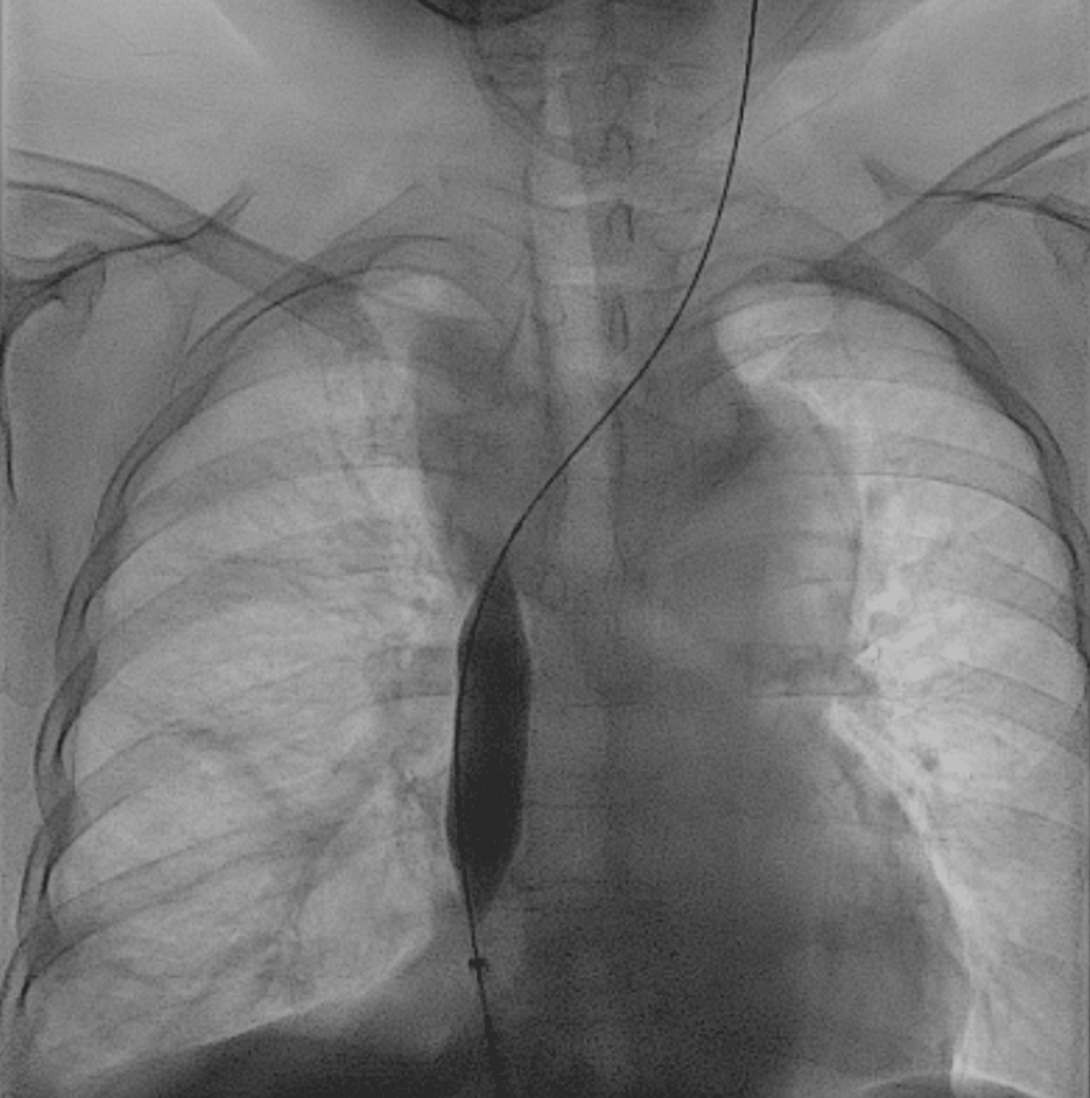
Balloon angioplasty of the superior vena cava

**Figure 5:**
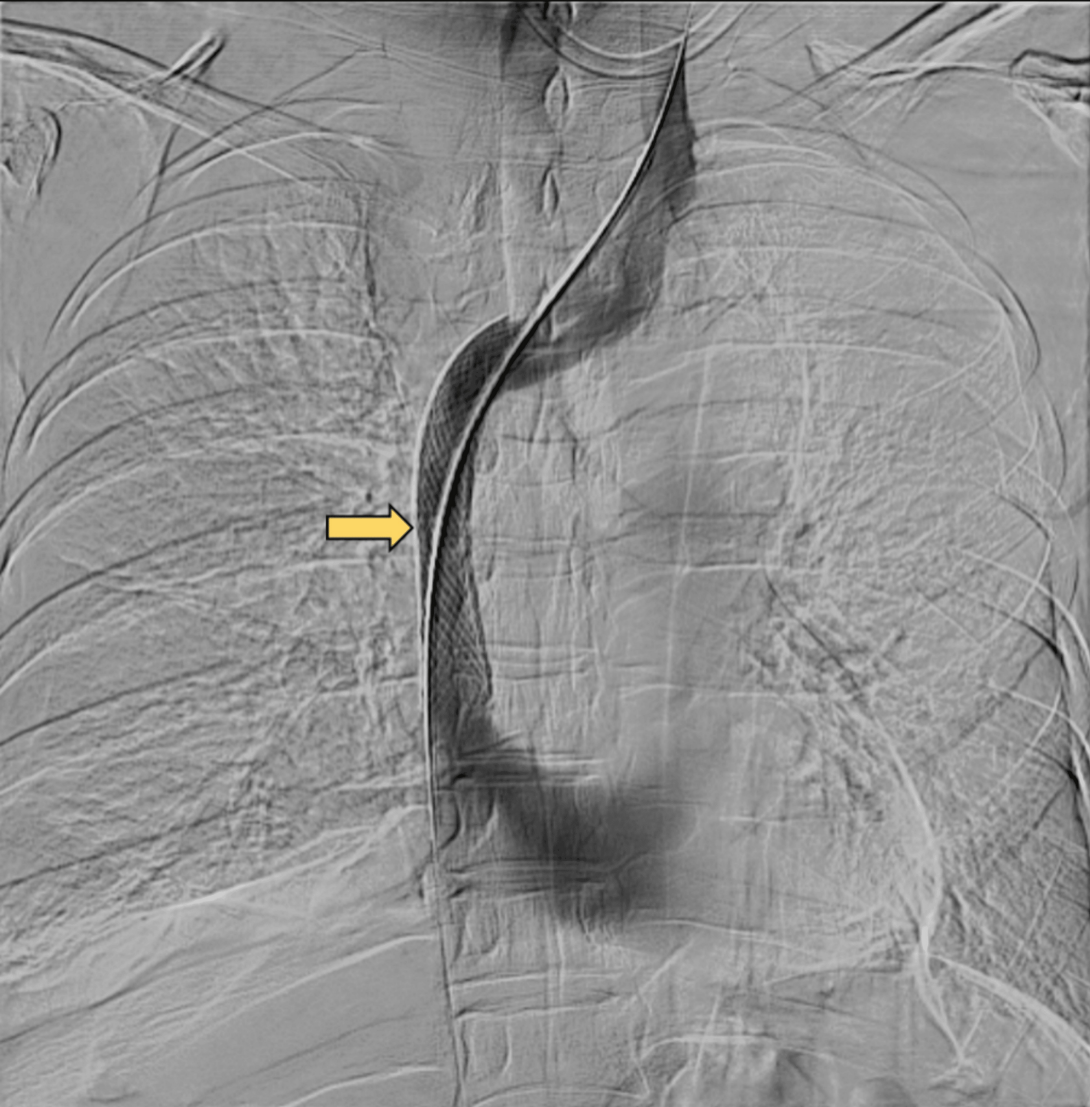
Final venogram post stenting demonstrating brisk antegrade flow across the superior vena cava stent (arrow) into the right atrium, with resolution of azygos vein collateralization.

Serial angioplasties were performed using the following balloons: 6 × 40 mm Mustang balloon (Boston Scientific, Marlborough, MA) for initial predilatation, 10 × 40 mm Mustang balloon (Boston Scientific) for intermediate dilatation, and 16 × 40 mm Atlas Gold high-pressure balloon (Becton Dickinson, Franklin Lakes, NJ) prior to stent placement (Figure 4). Due to significant recoil post-angioplasty, an 18 × 40 mm Wallstent (Boston Scientific) was deployed across the SVC, followed by post-stent balloon angioplasty with a 16 × 40 mm Atlas Gold balloon (Becton Dickinson). Final venography confirmed immediate restoration of venous patency and brisk antegrade flow into the right atrium (Figure 5).

**Figure 6:**
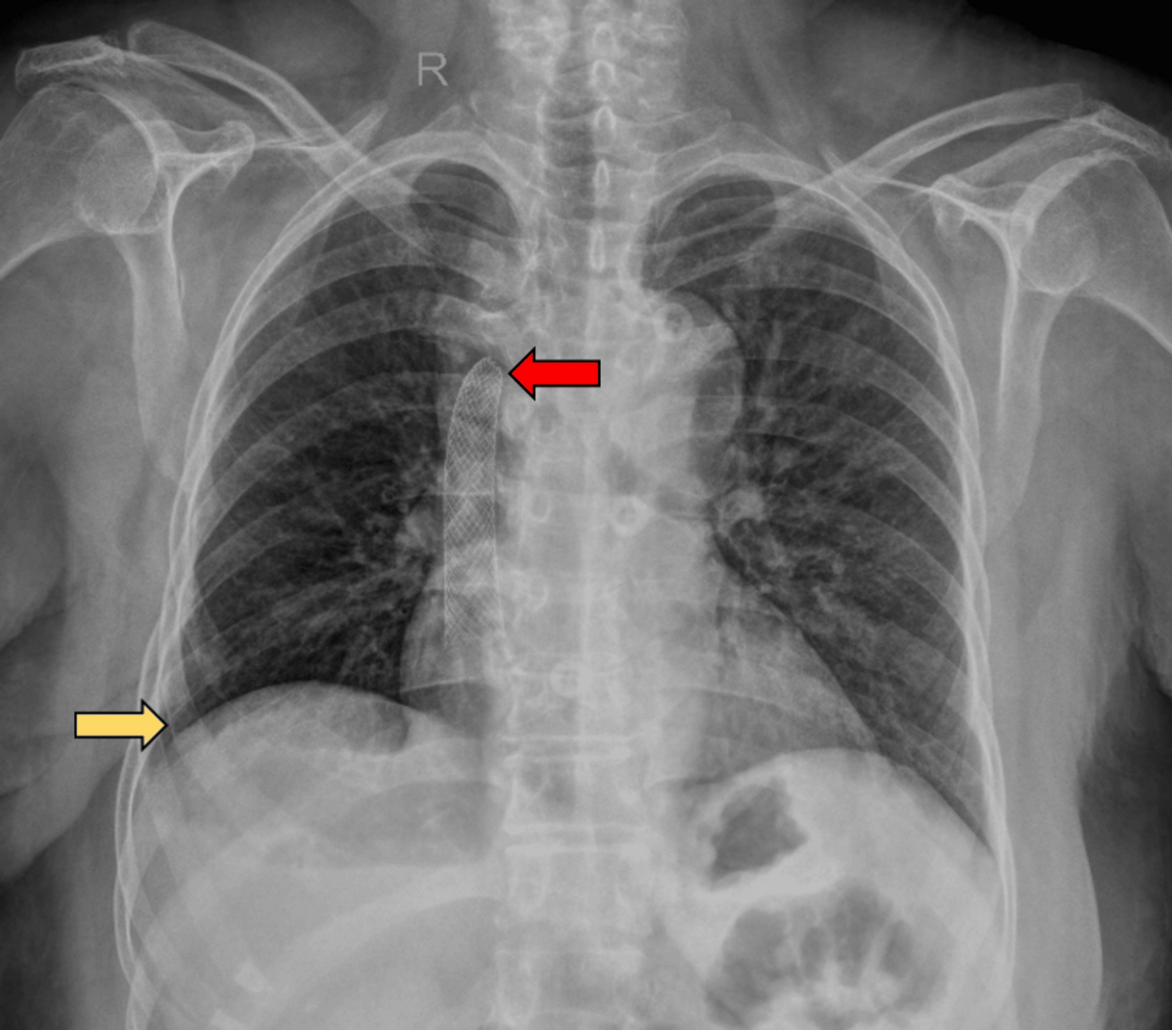
Post-procedural frontal chest radiograph showing complete resolution of pleural effusion (yellow arrow). Mild proximal stent pursing within the left brachiocephalic vein (red arrow), likely due to chronic venous fibrosis, was noted.

The patient tolerated the procedure well, with complete resolution of the chylothorax within 48 hours, as confirmed by post-procedural imaging. On the post-procedure chest X-ray, there was mild pursing of the stent at its proximal end within the left brachiocephalic vein, likely due to chronic venous fibrosis and reduced compliance. However, this did not impact the clinical response (Figure 6). She was discharged in stable condition on anticoagulation. No recurrence was noted on follow-up imaging at three months. 

## Discussion

Chylothorax is a relatively rare cause of pleural effusion, accounting for about 2% to 3% of cases. It results from the accumulation of lymphatic fluid in the pleural space due to injury or obstruction of the thoracic duct or its branches. The thoracic duct is the primary lymphatic vessel responsible for draining lymph from the lower body and the left upper body, usually emptying into the venous system at the junction of the left internal jugular and subclavian veins. When disrupted, chyle, a lipid-rich lymphatic fluid, leaks into the pleural cavity [1,2].

The etiologies of chylothorax are broadly divided into traumatic and nontraumatic causes. Traumatic injury to the thoracic duct and its tributaries is frequently iatrogenic, commonly occurring as complications of thoracic surgeries such as esophagectomy, cardiac surgery, or central venous catheterization [2]. Nontraumatic causes predominantly include malignancies, especially lymphomas, which may infiltrate or compress the thoracic duct. Other less common causes are granulomatous diseases like sarcoidosis, infections, amyloidosis, and vascular etiologies such as SVC obstruction [4,5].

SVC obstruction, though uncommon, is a significant cause of chylothorax, often manifesting on the right side. The pathophysiology involves increased hydrostatic pressure proximal to the obstruction site in the venous system, which is transmitted back to the lymphatic channels, impairing lymph drainage and causing chyle to accumulate in the pleural space. Unlike typical chylothorax caused by direct thoracic duct injury, those associated with SVC syndrome result from lymphatic hypertension due to impaired venous return [3].

Most chylothoraces are left-sided due to the anatomical drainage of the thoracic duct into the left venous angle. However, obstruction at the right internal jugular-subclavian junction or within the SVC itself can cause right-sided chylothorax, as observed in this patient. The presence of right-sided chylothorax should prompt clinicians to investigate central venous obstruction, especially in patients with histories of neglected indwelling vascular devices, malignancies, or prothrombotic conditions [6].

Clinically, chylothorax presents with symptoms such as dyspnea and chest discomfort. Nutritional and immunological deficits may occur due to the loss of lymphocytes and fat-soluble vitamins in the pleural fluid. Diagnosis requires pleural fluid analysis demonstrating a milky appearance, elevated triglycerides (>110 mg/dL), and the presence of chylomicrons. Imaging modalities such as CT venography or magnetic resonance (MR) lymphangiography are essential for identifying the underlying cause, such as venous obstruction or lymphatic leaks.

Management of chylothorax secondary to SVC obstruction focuses on correcting the venous blockage. Conservative approaches such as dietary modifications with medium-chain triglyceride diets or total parenteral nutrition, thoracentesis, and pleural drainage can relieve symptoms but do not address the primary pathology. Endovascular interventions, including angioplasty and stenting of the obstructed SVC, have emerged as effective, minimally invasive options to restore venous patency, reduce lymphatic pressure, and promote rapid resolution of the effusion [7]. In this case, successful recanalization and stenting of the chronically occluded SVC resulted in complete resolution of the chylothorax within 48 hours.

## Conclusions

This case highlights the importance of considering SVC syndrome in patients with unexplained chylothorax, especially right-sided. Early recognition and prompt endovascular intervention can significantly improve outcomes and prevent morbidity associated with persistent pleural effusions and more invasive surgical treatments.

Future studies are necessary to determine the long-term outcomes of SVC stenting in these cases and to establish standardized treatment guidelines. Moreover, advanced lymphatic imaging techniques such as dynamic contrast-enhanced MR lymphangiography and intranodal lymphangiography continue to evolve, potentially offering further insight into lymphatic and venous system pathophysiology.

## References

[1] Gomes AO, Ribeiro S, Neves J, Mendonça T (2015). Uncommon aetiologies of chylothorax: superior vena cava syndrome and thoracic aortic aneurysm. Clin Respir J.

[2] Rudrappa M, Paul M (2025). Chylothorax. https://www.ncbi.nlm.nih.gov/books/NBK459206/.

[3] Rice TW (2007). Pleural effusions in superior vena cava syndrome: prevalence, characteristics, and proposed pathophysiology. Curr Opin Pulm Med.

[4] de Villa AR, Obeidat O, Auyeung AB, Jaoude JA, Oyetoran A, Cannon K, Okonoboh P (2023). Superior vena cava syndrome presenting as chylothorax. Radiol Case Rep.

[5] Barracano R, Scognamiglio G, Palma M (2021). Chylothorax due to superior vena cava obstruction in a patient with complex congenital heart disease. JACC Case Rep.

[6] Itkin M, Kucharczuk JC, Kwak A, Trerotola SO, Kaiser LR (2010). Nonoperative thoracic duct embolization for traumatic thoracic duct leak: experience in 109 patients. J Thorac Cardiovasc Surg.

[7] Valentine VG, Raffin TA (1992). The management of chylothorax. Chest.

